# Relación entre el número de cepillados por día y la autopercepción periodontal en escolares ecuatorianos de 12 años

**DOI:** 10.21142/2523-2754-0901-2021-044

**Published:** 2021-03-11

**Authors:** Suley Elizabeth Castro-Cabrera, María Daniela Calle-Prado

**Affiliations:** 1 , Carrera de Odontología, Universidad Católica de Cuenca. Cuenca, Ecuador. zuleicka_0830@hotmail.com Universidad Católica de Cuenca Carrera de Odontología Universidad Católica de Cuenca Cuenca Ecuador zuleicka_0830@hotmail.com; 2 Carrera de Odontología, Universidad Católica de Cuenca. Cuenca, Ecuador. mcallep@ucacue.edu.ec Universidad Católica de Cuenca Carrera de Odontología Universidad Católica de Cuenca Cuenca Ecuador mcallep@ucacue.edu.ec

**Keywords:** cepillado dental, enfermedades periodontales, higiene bucal, Toothbrushing, Periodontal diseases, Oral hygiene

## Abstract

**Objetivo:**

Relacionar el número de cepillados por día y la autopercepción periodontal en escolares de 12 años de la parroquia Machángara, en Cuenca (Ecuador) en 2016.

**Materiales y métodos:**

Se realizó un estudio descriptivo, retrospectivo a nivel relacional. La muestra estuvo constituida por 205 fichas de escolares de 12 años residentes de la parroquia Machángara, que pertenecen al mapa epidemiológico de Salud Bucodental de Cuenca, realizado por la Universidad Católica de Cuenca. Para el análisis de los datos se empleó estadística descriptiva con frecuencias y porcentajes para cada una de las variables y la prueba estadística Tau-b de Kendall, con el fin de determinar la correlación entre las variables principales del estudio, según una significancia de 0,05.

**Resultados:** El 50,7% de los adolescentes fueron del sexo masculino; el 49,3%, del sexo femenino, y el 94,1% estudiaban en escuelas fiscales. Se halló que el 50,2% se cepillaban tres veces por día, frecuencia que se cumple en el 51,5% del sexo femenino y el 48,1% del sexo masculino. La prevalencia de autopercepción periodontal fue del 85,4%; de este total, el 86,1% correspondió a las mujeres y el 84,6% a los hombres. Se encontró una correlación negativa inversa y significativa entre el número de cepillados por día y la autopercepción periodontal (Tau-b: -0,178; p = 0,004). La frecuencia del cepillado presentó una correlación inversa y significativa con el autorreporte periodontal para el sexo femenino (tau-b: -0,197; valor p = 0,030) y no significativa en el sexo masculino.

**Conclusión:**

Existe una correlación negativa inversa y significativa entre la frecuencia del cepillado diario y la autopercepción periodontal. Es necesario aplicar estrategias educativas respecto de la salud oral en los adolescentes de la parroquia Machángara de Cuenca.

## Abstract (en)

**Objective:**

To determine the number of brushings per day and periodontal self-perception in 12-year-old schoolchildren from the Machángara parish, in Cuenca Ecuador in 2016.

**Materials and methods:**

A descriptive, retrospective, relational level study was conducted. The sample consisted of 205 records of 12-year-old schoolchildren residing in the Machángara parish, which belongs to the epidemiological map of oral health in Cuenca, performed by the Catholic University of Cuenca. For analysis of the data, descriptive statistics were used with frequencies and percentages for each of the variables and the Kendall Tau-b statistical test was performed to determine the correlation between the main variables of the study, according to a significance of 0.05.

**Results:**

50.7% of adolescents were male, 49.3% female, and 94.1% were in public schools. It was found that 50.2% of the study population brushed three times a day, being 51.5% among females and 48.1% among males. The prevalence of periodontal self-perception was 85.4%: 86.1% in girls and 84.6% in boys. An inverse and significant negative correlation was found between the number of brushings per day and periodontal self-perception (Tau-b: -0.178; p: 0.004). Brushing frequency had an inverse and significant negative correlation with periodontal self-reporting for females (tau-b: -0.197; p value: 0.030) and a non significant correlation for males.

**Conclusion:**

There is an inverse and significant negative correlation between the frequency of daily brushing and periodontal self-perception. Educational strategies regarding oral health are needed for adolescents from the Machángara parish of Cuenca.

## INTRODUCCIÓN

La salud es definida como un estado de equilibrio, ya sea físico, mental o social, y no simplemente como la ausencia de enfermedad o padecimientos, mientras que la salud oral es parte del estado de salud general de un individuo y resulta esencial para la calidad de vida ^1^. La enfermedad periodontal y la caries representan un alto porcentaje de las enfermedades bucales. La prevalencia de las enfermedades periodontales es de, al menos, un 40% a nivel general; no obstante, se considera que la gingivitis afecta al 80% de niños y adolescentes ^2^. Usualmente, las enfermedades orales están asociadas con los géneros bacterianos gramnegativos, como *Treponema, Bacteroides, Porphyromonas, Prevotella, Capnocytophaga, Peptostreptococcus, Fusobacterium, Actinobacillus* y *Eikenella*, presentes en la saliva, en el epitelio gingival y en otras superficies internas de la cavidad oral, concentrados en la placa dentobacteriana, por lo que es preciso llevar a cabo una higiene bucal adecuada y hacerlo a diario ^3^.

La higiene bucal incorrecta ocasiona la enfermedad periodontal, considerada una de las enfermedades bucales más frecuentes en los seres humanos ^(4, 5)^. Se trata de un mal infeccioso e inflamatorio causado por el mal control de la placa bacteriana, debido a la acumulación de biofilm, el cual, si no es eliminado oportunamente, genera la destrucción progresiva de los tejidos de soporte o la pérdida de las estructuras dentarias. Esta patología pone en riesgo el desarrollo de los individuos y afecta drásticamente su vida cotidiana al limitar sus funciones básicas ^6^.

Para eliminar completamente la placa bacteriana, lo esencial es su control y prevención a través de una limpieza adecuada. Esto se logra mediante el cepillado dental, el cual debe realizarse con una frecuencia de al menos 3 veces al día, para limpiar todas las piezas dentales, además de la lengua y las encías. El cepillado es la técnica más eficaz si se ejecuta de manera apropiada, pues conduce a una correcta higiene oral, sin embargo, debe complementarse con el uso de enjuagues bucales, hilo dental y pastas dentífricas para obtener mejores resultados ^7^^-^^9^.

Entre los principales problemas detectados durante el inicio y el progreso de la enfermedad periodontal se encuentra la gingivitis. Cuando esta no es tratada oportunamente, puede progresar a periodontitis y provocar desde la movilidad hasta la pérdida de los dientes. Las etapas iniciales de la enfermedad están asociadas con una mala higiene bucal, que no está relacionada con el estado socioeconómico ^10^.

En EE. UU., la enfermedad periodontal alcanza un rango del 40% al 60% en la población escolar y, según la Organización Panamericana de la Salud (OPS), en países de América Latina como el Perú y Colombia esta prevalencia aumenta hasta el 85%; en México, la prevalencia de las enfermedades periodontales se encuentra alrededor del 44% y en Chile es del 66,9% en los escolares. Por esto, la Organización Mundial de la Salud (OMS) considera la edad de 12 años como un momento importante para la vigilancia de enfermedades bucales y, además, se ha establecido como la edad de comparación internacional que ayuda al desarrollo de nuevas investigaciones ^11^^-^^13^. 

Por otra parte, la adolescencia es el período en el que la atención de la salud bucal se descuida más; por ello, la última encuesta del perfil periodontal realizada por la OMS, en 2016, demostró que el número de adolescentes con gingivitis aumentó sustancialmente en muchos países, con una mayor incidencia en los países menos desarrollados ^10^.

Los adolescentes, con frecuencia, experimentan problemas orales-dentales, predominantemente gingivitis, una condición inflamatoria de los tejidos que rodean el diente, debido a malos hábitos nutricionales, malos hábitos de autocuidado dental y también cambios hormonales en el momento de la pubertad ^1^. La enfermedad periodontal puede manifestarse a través del sangrado de las encías, lo cual se asocia generalmente con una mala salud oral. La presencia de sangre en las encías es causada por la formación de placa en la línea de las encías ^14^. La salud oral juega un papel importante en la definición del estado de salud general de un individuo y también para mantener una buena calidad de vida ^1^.

La autopercepción es un método eficaz y de gran acogida para la evaluación de características sobre la población, los factores de riesgo y las enfermedades, pero rara vez se ha utilizado para la enfermedad periodontal. Dado que uno de los factores de riesgo más comunes es la mala higiene bucal, relacionada con la presencia de la placa dentobacteriana, si esta no es removida tiende a acumularse y solidificarse, incluso puede formar cálculo supra y subgingival, y, en última instancia, al irritar e inflamar las encías, ocasionar que las bacterias y sus toxinas las infecten. Por tanto, obtener un informe reportado por el propio paciente sobre la presencia de signos o síntomas de la enfermedad periodontal resulta de gran utilidad para identificar la prevalencia en una población determinada y conocer la magnitud del problema, a fin de ofrecer atención y diseñar estrategias oportunas ^15^^-^^18^.

Dado que la autopercepción es una forma clara y concisa que ayuda a la estimación del estado de salud, al evaluar cómo los pacientes se sienten, piensan o perciben su estado de salud. La autopercepción estudia de forma indirecta las enfermedades periodontales percibidas por los escolares, lo cual quiere decir que el estudio se realiza a través de un cuestionario que mide la percepción propia de manera sencilla para obtener información útil con fines de intervención en materia de salud oral ^19^.

El objetivo general de esta investigación fue relacionar el número de cepillados por día y la autopercepción periodontal en escolares de 12 años de la parroquia Machángara, en Cuenca (Ecuador), en 2016, así como identificar el comportamiento de las variables según el sexo.

## METODOLOGÍA

Se realizó un estudio de tipo descriptivo y retrospectivo, en el cual se analizó la relación entre el número de cepillados diarios y la autopercepción periodontal sobre datos pertenecientes al estudio Mapa Epidemiológico de Salud Bucodental en escolares de la parroquia Machángara en 2016, desarrollado por la Carrera de Odontología de la Universidad Católica de Cuenca. 

La población objeto de este estudio estuvo conformada por 205 fichas de escolares de 12 años de la parroquia Machángara. Se incluyeron fichas que contaban con datos completos de escolares que participaron en la investigación y se aceptaron dos registros del sexo femenino que tenían datos faltantes en la variable frecuencia del cepillado diario, mientras que se excluyeron aquellas fichas que tuvieran más de una variable incompleta. La información contenida en la base de datos no permite identificar a los adolescentes participantes, por lo que se cumple con el principio de confidencialidad de los datos. 

El presente estudio no implicó conflictos bioéticos y fue revisado y aprobado por el Comité Institucional de Bioética en Investigación en Seres Humanos de la Universidad Católica de Cuenca. 

Las fichas fueron ingresadas en el programa Epi-Info 7, en las cuales se detallaba el sexo, el tipo de escuela, la frecuencia de cepillado por día y la autopercepción periodontal de cada escolar. Como criterios para establecer la frecuencia del número de cepillados, se utilizaron las siguientes categorías: menos de tres veces, tres veces y más de tres veces al día. Para establecer la autopercepción periodontal, se utilizó un cuestionario que constó de seis preguntas: 1. ¿Cree que tiene enfermedad de encías? 2. ¿Alguna vez le han hecho cirugía de encías? 3. ¿Alguna vez le han hecho un raspado de raíces dentales? 4. ¿Se le ha aflojado un diente no de leche? 5. ¿El dentista le ha dicho que ha perdido hueso? 6. ¿Le sangran las encías? Estas preguntas permitían a los escolares valorar su estado periodontal de manera subjetiva, considerándose como sano aquel que no reportara algún signo o síntoma de enfermedad periodontal.

**Figura 1:**
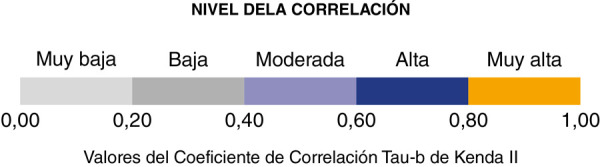
Criterios de evaluación del coeficiente de correlación Tau-b de Kendall^20^

Los datos fueron exportados al *software* estadístico SPPS, versión 25, en el que se procesaron las estadísticas descriptivas en forma de frecuencias y porcentajes, en tanto que la correlación entre las variables se calculó mediante el coeficiente de correlación Tau-b de Kendall y su significancia fue de 0,05. En la Figura 1 se presentan los criterios de evaluación del coeficiente de correlación Tau-b de Kendall, de acuerdo con su valor absoluto ^20^.

## RESULTADOS

Se evaluó a 205 escolares de 12 años, de los cuales 104 pertenecían al sexo masculino (50,7%) y 101, al sexo femenino (49,3%), con lo que se obtuvo una muestra equilibrada para cada sexo. Por otra parte, el 94,1% de la población pertenecía a colegios fiscales (Tabla 1).

**Tabla 1:** Distribución de la muestra de acuerdo al sexo y tipo de escuela

Variable	Categorías	n.^o^	%
Sexo	Femenino	101	49,3%
	Masculino	104	50,7%
	Total	205	100,0%
Tipo de escuela	Fiscal	193	94,1%
	Fiscomisional	12	5,9%
	Total	205	100,0%

En relación con la frecuencia de cepillado se observó que el 50,2% de los escolares de 12 años se cepillan tres veces al día, mientras que el 46,3% lo realiza menos de tres veces al día. En la base de datos se encontraron dos valores perdidos para esta variable, lo cual representó apenas el 1% del total de registros. Se pudo apreciar que el 52,5% de las mujeres se cepillan tres veces al día, mientras que en los hombres esta frecuencia de cepillado la realiza el 48,1%; sin embargo, estos porcentajes casi son equiparados por la frecuencia de cepillado de menos de tres veces diarias, ya que en las mujeres alcanza el 46,5% y en los hombres, el 46,2% (Tabla 2).

**Tabla 2:** Frecuencia de cepillado al día en la parroquia y de acuerdo con el sexo

Sexo	Veces de cepillado al día	n.^o^	%
Total	Menos de 3 veces al día	94	46,3%
	Tres veces al día	102	50,2%
	Más de 3 veces al día	7	3,4%
	Total	203	100,0%
	Valores perdidos	2	
	Total	205	
Femenino	Menos de 3 veces al día	46	46,5%
	Tres veces al día	52	52,5%
	Más de 3 veces al día	1	1,0%
	Total	99	100,0%
	Valores perdidos	2	
	Total	101	
Masculino	Menos de 3 veces al día	48	46,2%
	Tres veces al día	50	48,1%
	Más de 3 veces al día	6	5,8%
	Total	104	100,0%

El 14,6% de los escolares de la muestra reportan un estado sano de su salud bucal, por lo que el 85,4% restante reporta algún signo o síntoma de enfermedad periodontal. Asimismo, se puede apreciar que el 86,1% de las mujeres y el 84,6% de los hombres reportan signos o síntomas de enfermedad periodontal (Tabla 3).

**Tabla 3:** Autopercepción periodontal general y según el sexo

Sexo	Puntaje del autorreporte periodontal	n.^o^	%
Total	0	30	14,6%
	1	65	31,7%
	2	51	24,9%
	3	40	19,5%
	4	14	6,8%
	5	4	2,0%
	6	1	0,5%
	Total	205	100,0%
Femenino	0	14	13,9%
	1	31	30,7%
	2	26	25,7%
	3	20	19,8%
	4	8	7,9%
	5	1	1,0%
	6	1	1,0%
	Total	101	100,0%
Masculino	0	16	15,4%
	1	34	32,7%
	2	25	24,0%
	3	20	19,2%
	4	6	5,8%
	5	3	2,9%
	6	0	0,0%
	Total	104	100,0%

**Figura 2:**
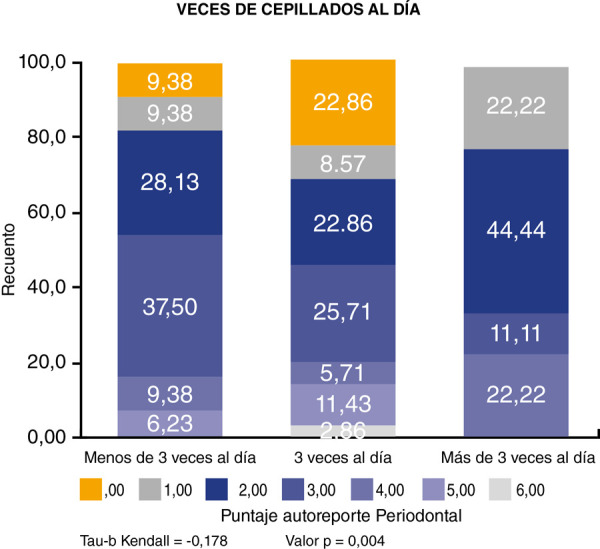
Correlación entre el número de cepillado por día y autopercepción periodontal

Al calcular la correlación mediante el coeficiente Tau-b de Kendall, su resultado fue de -0,178, indicando una relación negativa e inversa entre la frecuencia del cepillado dental y el autorreporte periodontal; esto quiere decir que a mayor número de cepillados por día menor será el valor de autopercepción periodontal en los adolescentes. Asimismo, se puede apreciar que dicha correlación es significativa entre las dos variables, dado que el valor p de significancia fue de 0,004, inferior a 0,05 (Figura 2).

**Figura 3:**
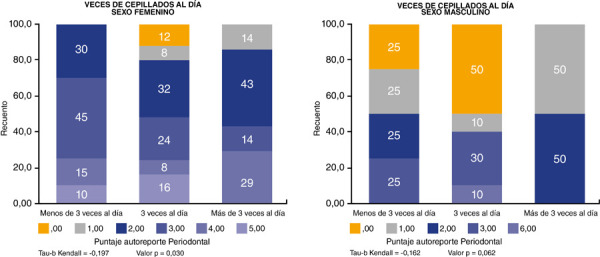
Correlación entre el número de cepillado por día y autopercepción periodontal por sexo

Continuando con el análisis de correlación, se tiene la distribución del puntaje de autorreporte periodontal según la frecuencia del cepillado para cada sexo (figura 2). Para el sexo femenino existe una correlación negativa inversa entre la frecuencia del cepillado diario y autopercepción periodontal, ya que el coeficiente Tau-b de Kendall es de -0,197; se puede interpretar que, a mayor frecuencia de cepillado dental por día menor será el valor de la autopercepción periodontal en las mujeres. Esta correlación es significativa, puesto que el valor p es de 0,030, inferior al nivel de significancia establecido de 0,05. Asimismo, para el sexo masculino, se tiene que también existe una correlación negativa e inversa entre las dos variables, puesto que el coeficiente Tau-b de Kendall es de -0,162; sin embargo, esta correlación no es significativa, ya que el valor p es de 0,062, mayor a 0,05 (Figura 3).

## DISCUSIÓN

La presente investigación fue realizada en la parroquia Machángara del cantón Cuenca (Ecuador) para determinar la relación entre la frecuencia del cepillado diario y la autopercepción periodontal en adolescentes escolares de 12 años. La investigación se llevó a cabo mediante un análisis descriptivo y el coeficiente de correlación Tau-b de Kendall. La muestra estuvo conformada por 205 escolares, de los cuales el 50,7% fueron del sexo masculino y 49,3%, del femenino, con lo que obtuvo una muestra equilibrada por sexo; mientras que el 94,1% de los escolares pertenecía a escuelas fiscales o públicas. En el estudio realizado por Alvear-Ordóñez et al. ^21^ en la parroquia Gil Ramírez Dávalos de Cuenca, la proporción de adolescentes femeninas fue del 71,0% y el 29% de masculinos, por lo que presentó una relación completamente diferente a la de este estudio. Por su parte, en el estudio publicado por Naranjo-Rodríguez et al. ^22^ en la parroquia Chiquintad de Cuenca las proporciones por sexo fueron del 53% en el femenino y el 47% en el masculino.

Del total de adolescentes de 12 años de la investigación, el 50,2% se cepillaban tres veces al día y el 46,3% menos de tres veces; además, el 52,5% de las mujeres y el 48,1% de los hombres se cepillaban tres veces al día. Datos similares fueron los reportados en escolares de una población en México por Arrieta et al. ^23^, en cuyo caso el 61% cepillaba sus dientes tres veces al día. Naranjo-Rodríguez et al. ^22^ analizaron la frecuencia del cepillado en adolescentes escolares de 12 años de la parroquia Chiquintad de Cuenca y observaron que el 49% se cepillaban tres veces al día, mientras que el 46% lo realizaba menos de tres veces al día. Estos autores también realizaron una distribución de la frecuencia del cepillado por sexo y hallaron que un 56,6% de las mujeres y un 40,4% de los hombres se cepillan tres veces al día, en tanto que el 39,6% de las mujeres y el 53,2% de los hombres se cepillaban menos de tres veces al día, lo cual es levemente contradictorio a lo encontrado en este estudio. 

En el estudio realizado por García et al. ^24^ en el Perú, reportan valores del 34,3% que cumplen con una frecuencia del cepillado de dos veces al día, visiblemente inferior a lo reportado en este estudio. Por su parte, en la investigación de Costa et al. ^25^, realizada en el municipio de São Lourenço da Mata, en Pernambuco (Brasil), se encontró que el 40% de los adolescentes cepillaban sus dientes una sola vez por día, lo que representa un factor de riesgo alto e importante para la salud bucal.

El 14,6% de los escolares reportó un estado sano de su salud bucal, por lo que el 85,4% señaló algún signo y síntoma de enfermedad periodontal; asimismo, el 86,1% de las mujeres y el 84,6% de los hombres reportaron signos o síntomas de enfermedad periodontal. Alvear Ordóñez et al. ^21^ encontraron que, en la población escolar de 12 años de la parroquia Gil Ramírez Dávalos de Cuenca, el 81% presentaba manifestaciones de la enfermedad periodontal, mientras que por sexo estas características se presentaron en el 82% de las mujeres y el 83% de los hombres. Por otra parte, Mohd Dom et al. ^26^ realizaron un estudio sobre las prácticas de higiene oral autoinformadas y el autorreporte del estado periodontal, en el cual encontraron que un 81% de los participantes de la muestra detectaron gingivitis y otros signos de enfermedad periodontal. 

La correlación entre la frecuencia del cepillado dental y el autorreporte periodontal fue negativa e inversa (tau-b: -0,178), lo cual indica que a mayor número de cepillados por día menor es el índice de autopercepción periodontal en los escolares de 12 años de la parroquia Machángara de Cuenca, relación que además es significativa (p = 0,004). Esto también se cumple para el sexo femenino, en cuyo caso el Tau-b de Kendall mostró un valor de -0,197 y un p = 0,030, pero no en el caso del sexo masculino, que mostró un p = 0,062. Por el contrario, en el estudio de Serrano, Niño y Romero ^27^, realizado en la ciudad de Bogotá (Colombia), no se encontró asociación significativa entre la frecuencia del cepillado diario y la enfermedad periodontal, mientras que acudir a consulta odontológica sí fue relevante para la presencia o ausencia de enfermedad periodontal. Asimismo, en otra investigación realizada en Madrid (España) por Járitzon et al. ^28^ no se encontró relación estadística significativa entre la frecuencia del cepillado y la presencia de enfermedad periodontal.

Entre las fortalezas encontradas en este estudio se puede mencionar la facilidad con que el instrumento para el autorreporte puede ser aplicado, la practicidad con la cual se recopila la información y la rapidez con la que se obtiene un indicador de la frecuencia de autorreporte de la enfermedad periodontal; aunque este tipo de instrumentos autoaplicados también puede representar un sesgo en la respuesta del escolar y presentar variaciones no intencionadas, debido a las diferencias en la comprensión que pueda tener un escolar y respecto de lo que debe evaluar sobre la presencia de signos o síntomas de la enfermedad periodontal. 

Por otra parte, se observaron algunas debilidades, tales como un tamaño de muestra relativamente bajo, así como un mínimo número de variables que no permitió profundizar en los factores de riesgo relacionados con la enfermedad periodontal en los adolescentes, tales como el número de visitas al odontólogo, la técnica del cepillado dental, el estado del cepillo de dientes, el uso de crema e hilo dental, y el nivel socioeconómico de la familia. En futuras investigaciones deberían considerarse variables que complementen el análisis y permitan indagar aún más sobre el problema de la enfermedad periodontal en los adolescentes.

## CONCLUSIONES

Existe una correlación negativa e inversa entre el número de cepillados diarios y la autopercepción periodontal, es decir, a mayor frecuencia de cepillado los valores de autopercepción periodontal disminuyen, lo cual se observa con mayor énfasis en el sexo femenino.

## Fuente de financiamiento:

Autofinanciado
